# Patient satisfaction and quality of life outcomes following robotic-assisted surgery: A survey-based study

**DOI:** 10.6026/9732063002001964

**Published:** 2024-12-31

**Authors:** Keerthika Muniasamy, Anisha Sivakumar, Kaushik Nattamai Rameshbabu, Aswin Ganesan, Vibhiksha Arun Rajkumar, Gaurav Vijayrao Deshmukh, Sanjana S.V., Shivam Sharma

**Affiliations:** 1 Department of Surgery, Madras Medical College, Chennai, India; 2 Department of General Surgery, Madras Medical College, Chennai, India; 3 Institute of Internal Medicine, Madras Medical College, Chennai, India; 4 Department of Medicine, Thanjavur Medical College, Thanjavur, India; 5 Department of SNCU, District Hospital, Ahmednagar, Maharashtra, India; 6 Institute of Internal Medicine, Madras Medical College, Chennai, India; 7 Department of Surgery, Guru Gobind Singh Medical College, Faridkot, Punjab, India

**Keywords:** Robotic-assisted surgery, patient satisfaction, quality of life, postoperative outcomes, survey-based study

## Abstract

Robotic-assisted surgery has gained interest due to its potential for improved precision, reduced trauma and quicker recovery. This cross-sectional survey assessed patient satisfaction and quality-of-life outcomes in 100 patients who underwent robotic-assisted procedures across various specialties. The findings revealed high satisfaction levels, with 85% of patients expressing positive feedback about surgical outcomes. Quality-of-life improvements were noted in pain reduction, physical recovery and psychological well-being. Minor dissatisfaction arose from discomfort during postoperative stages and extended recovery periods in complex cases. The results highlight the need for enhanced preoperative counseling to align patient expectations, reinforcing robotic-assisted surgery as a method associated with high satisfaction and improved quality of life.

## Background:

It has been two decades of continuous rapid evolution of robotic-assisted surgery, bringing a new transformative approach to a wide variety of surgical interventions. With this development of technology, there has increasingly been the use of robotic systems, such as the da Vinci Surgical System, across specialties, notably in urology, gynecology and cardiothoracic surgery [1]. Robotic-assisted procedures are, in most respects, associated with increased precision, flexibility and control, thus often reflecting better outcomes, such as lower blood loss, smaller incision size and a shorter length of hospital stay [2]. These benefits are most important in complex cases where incredibly careful precision is of importance; therefore, it becomes incredibly attractive for surgeons and patients alike [3]. Another important metric for measuring outcomes of surgical procedures is patient satisfaction and quality of life. The outcome assessment evaluates not only the success of the procedure but also the general well-being of the patient and the level of satisfaction with care. Previous studies have indicated that the patient satisfaction rates may be higher after robotic-assisted surgeries as compared to other traditional methods, mainly because the patients recover more quickly, report less post-operative pain and have better functional outcomes [4]. For instance, patients undergoing robotic-assisted prostatectomy have demonstrated superior urinary and sexual function post-surgery over open or laparoscopic approaches. That would improve quality of life post-surgical intervention [5]. Similarly, pain and recovery profiles in robotic-assisted gynaecologic oncology procedures have been favourable and are key contributory factors for the quality of life post-treatment [6]. Robotic-assisted surgery has its own set of challenges though. Excessive cost of robotic systems and even resources to introduce them often cause difficulties in accessing robotic-assisted surgery and reduce its availability in some healthcare settings [7]. Patients may also come into surgery with heightened expectations due to perceived technological advancement associated with surgical robotics and experience disappointment if outcomes do not live up to such expectations [8]. Similar technical challenges arise due to the lack of direct tactile feedback, inherent in robotic systems, which can interfere with postoperative recovery and patient experiences in some complex cases [9]. With the increasingly common use of robotic-assisted surgery, knowledge about patient satisfaction and quality of life outcomes can judge the true value of these procedures. While numerous studies up to date have concentrated on clinical results, such as complication rates and recovery times, few have focused on patient-reported outcomes and satisfaction. The objective of this study is to assess patients' perception of quality of life and satisfaction following robotic-assisted surgery across different specialties, identifying areas of interest and factors that may influence patient perceptions and thus lead to improving patient education and preoperative counselling. Therefore, it is of interest in presenting insight that could shed light on how robotic surgery may influence patients' lives beyond clinical assessment and therefore contribute to having a more holistic view of the procedure's overall effectiveness [10].

## Methodology:

The current study describes a cross-sectional survey assessment of patient satisfaction and quality-of-life outcomes after robotic surgery compared across surgical specialties, including urology, gynaecology and general surgery. It includes patients who underwent robotic-assisted surgery in the last 12 months to be sure that enough time has passed for recovery as well as to ensure the maintained postoperative results in a stable state. The sample comprised 100 patients selected from different hospital settings who possessed the following selection criteria to be chosen: those patients whose age is above 18 years old and with the ability to give competent consent and to answer the survey independently. The questionnaire was formulated based on literature review with consultation of the surgeons and clinical psychologists dealing with patients' post-surgery. These include demographic and clinical characteristics, general satisfaction of the results of surgery, perceived quality-of-life changes by the patients and factors that would affect the level of satisfaction. The quality of life questionnaire consisted of questions related to physical functioning and emotional well-being and the level of satisfaction was measured on a 5-point Likert scale, with 1 indicating very dissatisfied and 5 indicating very satisfied. The overall experience of surgery has been addressed through targeted questionnaires focused specifically on speed of recovery, postoperative pain and perceived benefits from the surgery. Open-ended questions were tacked on to allow patients to note any specific challenges or unexpected results they may have encountered. The survey was administered in person at follow-up visits and via secure email links soliciting individual preferences. It was collected over a period of two months with reminders every two weeks to encourage more responses. It was volunteered and, above all, the responses kept anonymous to help create honesty in the survey. For demographic variables and responses to the survey, means, standard deviations and frequency distributions were calculated. To determine the existence or otherwise of any differences regarding outcomes in terms of satisfaction and quality of life based on age group, gender, or type of surgical procedure, t-tests and ANOVA tests will be used. Data analysis will be performed using SPSS software version 25.0, at a level of statistical significance of p < 0.05. Ethical clearance was sought from an Institutional review board where all participants were informed and agreed upon their participation in the research through informed consent. The participants' confidentiality and anonymity were ensured during the period of the study through keeping the data safely that could only be accessed by authorized personnel in research.

## Questionnaire:

This questionnaire was divided into subsections to ensure that more complete data were collected on the demographics of the patients, satisfaction with the outcomes of surgery, effectiveness of management of postoperative pain, experience of recovery and changes in quality of life from the surgery. Each section aimed to cover objective as well as subjective information about patients to enable an in-depth assessment of patient perception. There were open-ended questions to enable giving space for expatiations by the patients on their experiences with identification of particular aspects of the postoperative journey which had impacts on their general satisfaction or dissatisfaction.

## Table 1 below shows the summary of the questionnaire structure:

The questionnaire collected information on demographics, surgical outcomes, pain management, recovery and quality of life, with open-ended questions that allowed patients to elaborate on key aspects influencing their satisfaction.

## Results:

Table 2 provides an overview of participant demographics, including age, gender and type of robotic-assisted surgery undergone. Table 3 summarizes patient satisfaction levels regarding the overall success of the robotic-assisted surgery. Table 4 indicates patient perceptions of pain management effectiveness, highlighting areas where some patients reported suboptimal pain control. Table 5 presents the reported recovery times, with the majority of patients noting a relatively quick return to daily activities. Table 6 highlights the percentage of patients reporting improvements in their quality of life post-surgery. Table 7 reflects physical well-being changes reported by patients after RAS. Table 8 captures emotional well-being changes, indicating most patients felt positive emotional outcomes after RAS. Table 9 presents patient feedback on the ease of resuming daily activities post-surgery. Table 10 shows the alignment between patients' preoperative expectations and their actual outcomes after surgery. Table 11 highlights themes from open-ended responses, with patients mentioning effective communication and follow-up care as key to satisfaction.

## Discussion:

The aim of this study was to evaluate post-robotic surgical satisfaction and quality of life among various specialties. These findings are given based on general feedback by patients regarding their overall satisfaction and effective quality of life that changed post-surgery. In addition, these results further add to an ever-increasing number of findings that establish evidence of advantages created by robotic-assisted surgical techniques. Most patients in this study were satisfied with their surgical outcome and 85% of them would render positive feed on their procedure. This would not be any different from previous studies that have documented high patient satisfaction rates associated with robotic-assisted surgery owing to factors such as reduced post-operative pain, minimum scarring and quicker return to daily activities [11]. Robotic systems may result in better surgical outcomes due to enhanced precision and control, thus fulfilling greater expectations from patients. However, a proportion of the patients were slightly dissatisfied and complaints mainly referred to the unexpected postoperative pain and longer-than-anticipated recovery times in complex cases [12]. Such dissatisfactions seem to necessitate optimal preoperative counselling regarding the likely postoperative course. In this, clear and specific information regarding the risk and recovery profile useful in helping the patient set realistic expectations from their care providers. Providing conditions that need improvement contributes positively toward increasing the quality of life, especially considering physical functioning, alleviated pain and emotional well-being. Such findings are in congruence with literature that suggests that robotic-assisted surgery may result in better functional outcomes and improved quality of life after operation compared to the conventional surgeries [13]. For instance, patients who have had their prostates taken out through robotic prostatectomy reported marked improvement in urinary and sexual function, highly interfering with their quality of life. The minimal invasiveness of robotic-assisted surgery has also attributed to fewer traumas to the body, less hospital stays and shorter recovery periods [14]. Such fast recovery enables patients to quickly get back to their routine activities that positively impact their psychological status with better quality life. Moreover, the accuracy of movement by instruments can reduce complications and better preserve healthy tissue during the surgical procedure, which enhances postoperative quality of life. Though the general results were positive, there could be other facts that might affect the patient satisfaction and quality of life. The type of surgery, age of the patient, comorbidities he had and the surgeon's experience with robotic systems may also play a role in the outcome [15]. For example, an older patient or someone with major comorbidities may have a longer recovery period or complications that may compromise improvements in patient satisfaction and quality of life. Training time may also represent another possible surgeon-related aspect that could impact the effectiveness and outcomes of surgery. Experienced surgeons who use robotic technology most often tends to find surgeries easier, not only because they spend more time with the technology but also because they get used to using it more frequently [16].

It further highlights that proper training and credentialing must be undertaken to ensure a surgeon is adequately exposed to robotic systems for them to use appropriately. The benefits presented in this study concerning the positive results of robotic-assisted surgery provides evidence that it may be a better alternative for surgeries performed otherwise almost impossible or challenging through either open or laparoscopic surgery. Earlier studies have concluded that, overall, robotic surgery has had less blood loss during the surgery, fewer complications and much shorter hospital stays [17]. These benefits also translate to increased patient satisfaction and quality of life, as our data also indicate. Importantly, however, robotic-assisted surgery is not appropriate for all patients or procedures. The choice of the surgical technique should be individualized according to characteristics of the patients, expertise of surgeons and allocated resources [18]. In some instances, traditional surgical techniques can be equivalent or even better in achieving the same outcomes, especially if robotic technology is not available or in the hands of a surgeon who prefers traditional techniques. Several limitations are noted regarding the nature of this study. The sample size being 100 patients, though sufficient for pilot study results, limits generalization. Further, the cross-sectional design does not capture only the patient's perception at one point in time but does not include changes in satisfaction and quality of life over time. Recall bias may also occur, where patients forget to recall the state or facts before surgery or the recovery process. By using self-report satisfaction and quality-of-life measures that are susceptible to interference by patient expectations and subjective perceptions, the study conducted no objective outcome measurements [19]. Longitudinal designs combining objective outcome measures might offer more comprehensive insights into the long-term effects of robotic-assisted surgery on patient satisfaction and quality of life. Ideally, future studies should be large and multicentre to better generalize results. Identification of specific surgical procedures with separate handling might be able to identify what kind of surgery benefits most from robot-assisted surgeries. Another way to determine cost-effectiveness would be within the patient perspective and healthcare system outcomes since robotic systems are extremely expensive [20]. Standardized patient education programs would also further enhance patient satisfaction if established to manage realistic expectations regarding the surgical result and the course of recovery. Researching on the development and effectiveness of such programs would prove useful. Surgeon training and assessment with robotic systems should also be on-going for an extremely elevated level of proficiency and optimal results in patients. In the early phase of recovery, patients who had TLH reported significantly greater improvement in QoL from baseline compared with those who had TAH, in all subscales apart from emotional and social wellbeing. Improvements in QoL up to 6 months after surgery continued to favour TLH, except in the emotional and social wellbeing measures of FACT and the visual analogue scale of the EuroQoL five dimensions (EuroQoL-VAS) [21]. Although some studies demonstrated no significant difference between the cohorts, most of the evaluated studies demonstrated a shorter recovery time to reach baseline or better QOL in patients who underwent TORS [22]. The majority of women, both young and old, required either no analgesics or only non-steroidal anti-inflammatory agents for pain control [23]. There are well-described benefits to minimally invasive surgery including decreased blood loss, shorter hospital-stay and faster recovery. The role of robotic surgery in gynaecologic oncology has become increasingly prominent. It is uncertain to what degree this resulted from simply having undergone surgery as opposed to benefits unique to the surgical approach [24].

## Conclusion:

Patients' satisfaction and quality of life after robotics surgery are generally high. Reduced pain, faster recovery and better physical and psychological well-being usually begin to manifest after surgery. However, proper grounding of expectations and continued support enhance patient experience and outcome. Providers can improve patient-centered care for patients by focusing on these issues and ensure that patients experience the best potential in robotic-assisted surgery.

## Figures and Tables

**Table 1:** Summary of the Questionnaire

**Section**	**Focus Area**	**Details Included**
Demographics	Background information	Age, gender, type of surgery, time since surgery
Satisfaction with Surgical Outcomes	Patient's assessment of surgery success	Satisfaction with surgical results, perceived benefits of RAS
Pain Management	Postoperative pain experiences	Pain levels during recovery, effectiveness of pain management strategies
Recovery Experience	Recovery time and complications	Length of recovery, any complications experienced, ease of returning to daily activities
Quality of Life	Overall well-being post-surgery	Physical, emotional and social well-being changes after surgery
Open-Ended Responses	Additional patient insights	Any specific challenges or feedback related to RAS experience

**Table 2:** Demographic characteristics of respondents

**Variable**	**Percentage (%)**
Age 18-34	20
Age 35-50	30
Age 51-65	35
Age 66+	15
Male	55
Female	45
Urological Surgery	40
Gynaecological Surgery	30
General Surgery	30

**Table 3:** Satisfaction with surgical outcomes

**Satisfaction Level**	**Percentage (%)**
Very Satisfied	50
Satisfied	35
Neutral	10
Dissatisfied	5

**Table 4:** Pain management effectiveness

**Pain Management Rating**	**Percentage (%)**
Highly Effective	45
Effective	40
Neutral	10
Ineffective	5

**Table 5:** Recovery time post-surgery

**Recovery Duration**	**Percentage (%)**
Less than 2 weeks	30
2-4 weeks	45
More than 4 weeks	25

**Table 6:** Quality of life improvement

**Quality of Life Change**	**Percentage (%)**
Significant Improvement	55
Moderate Improvement	30
No Change	10
Decrease in Quality	5

**Table 7:** Physical well-being post-surgery

**Physical Well-Being**	**Percentage (%)**
Improved	60
No Change	30
Worsened	10

**Table 8:** Emotional well-being post-surgery

**Emotional Well-Being**	**Percentage (%)**
Improved	50
No Change	40
Worsened	10

**Table 9:** Ease of returning to daily activities

**Ease of Return**	**Percentage (%)**
Very Easy	45
Easy	35
Neutral	15
Difficult	5

**Table 10:** Patient expectations vs. outcomes

**Alignment Level**	**Percentage (%)**
Exceeded Expectations	25
Met Expectations	55
Below Expectations	20

**Table 11:** Common themes from open-ended responses

**Theme**	**Percentage of Responses (%)**
Effective Communication	60
Follow-Up Care	50
Expectations Management	40
